# Supplementary material for: Multimodal analysis of RNA sequencing data powers discovery of complex trait genetics
Source: Nat Commun. 2024 Nov 29;15:10387. doi: 10.1038/s41467-024-54840-8 (PMC11607376; doi:10.1038/s41467-024-54840-8)
Supplement: Supplementary file 1 — Supplementary Information [file 41467_2024_54840_MOESM1_ESM.pdf]

Supplementary Information for

Multimodal analysis of RNA sequencing data powers discovery of complex trait genetics

Included in this file:
Supplementary Figures 1-9
Supplementary Methods
Supplementary References

Supplementary Figures

Supplementary Figure 1: RNA phenotypes and represented genes per GTEx tissue.

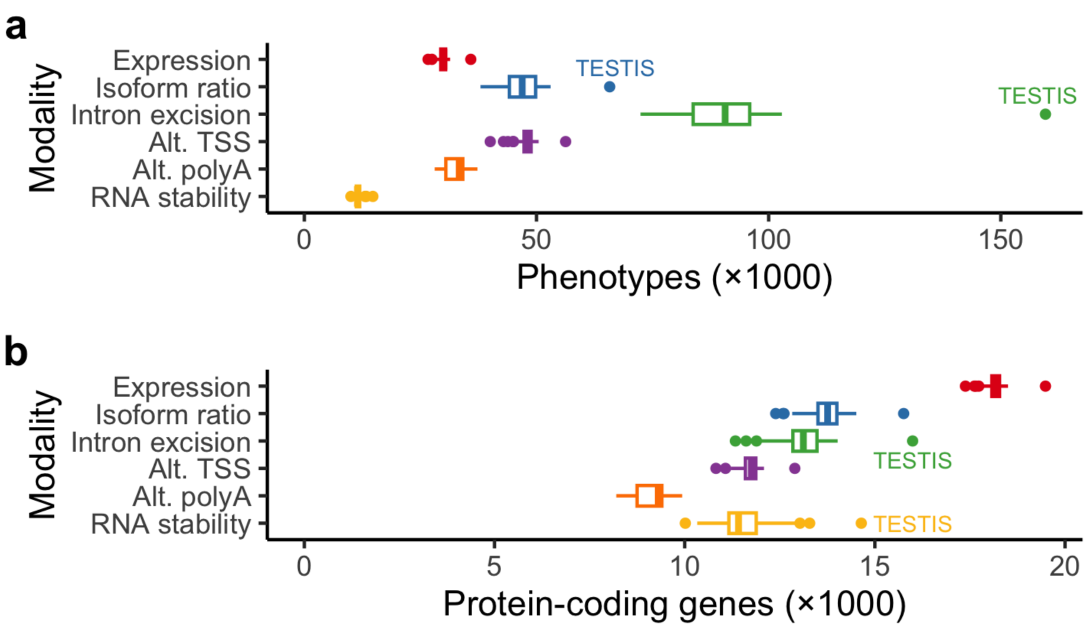

a boxplots showing the number of phenotypes extracted per GTEx tissue per modality, for all protein-coding genes and lncRNAs. Boxplots are colored by modality. **b** The number of protein-coding genes represented by the phenotypes above. Source data are provided as a Source Data file.

Supplementary Figure 2: Expression and non-expression xQTLs in GTEx tissues.

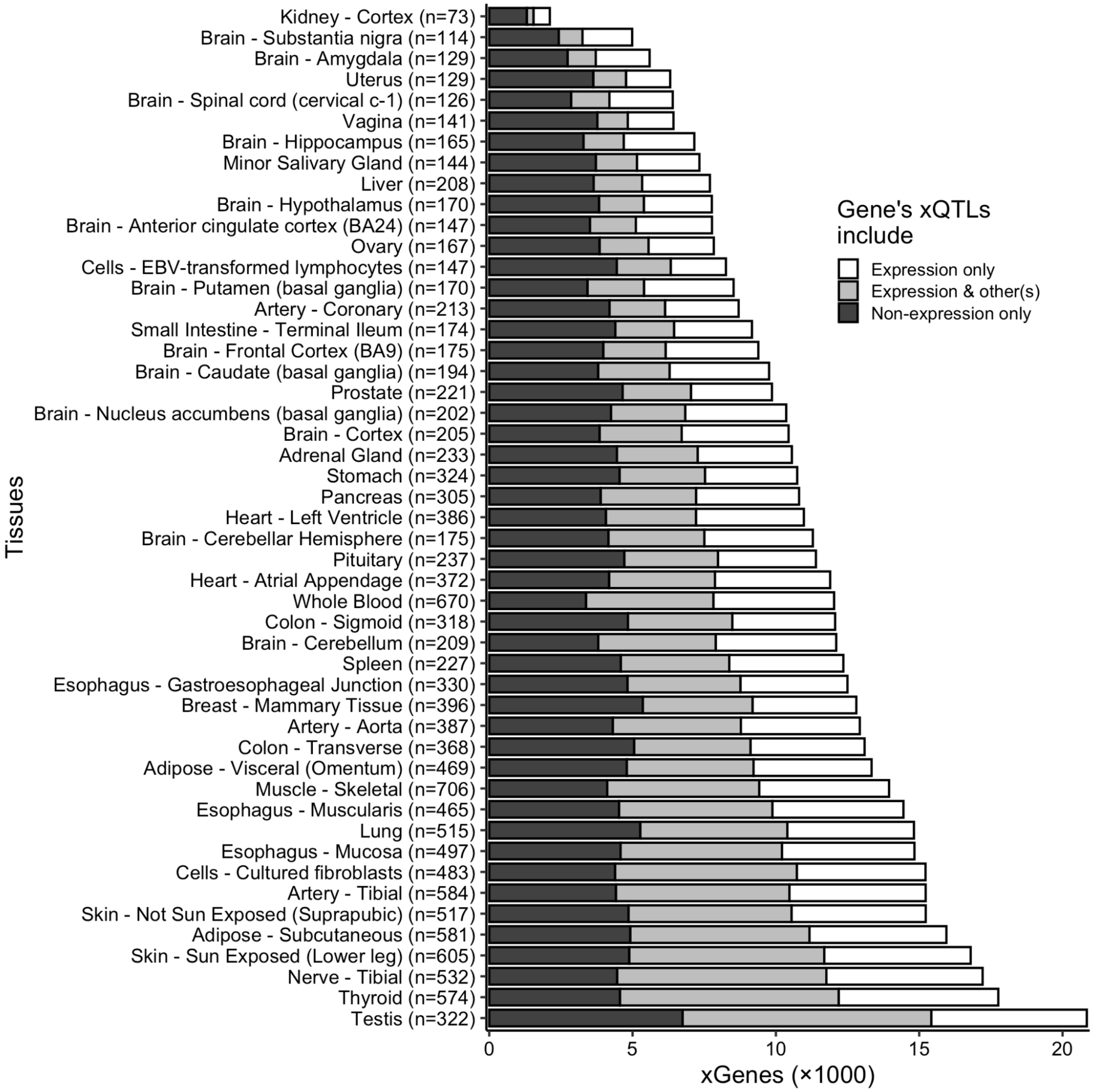

For each GTEx tissue, the number of genes with at least one xQTL are shown, colored by whether each gene's xQTL(s) included eQTLs, one or more other modality of xQTL, or both. Results are shown for xQTLs mapped separately per modality to show the increase in gene count compared to eQTL mapping alone. Source data are provided as a Source Data file.

Supplementary Figure 3: Concordance of xQTLs between independent datasets.

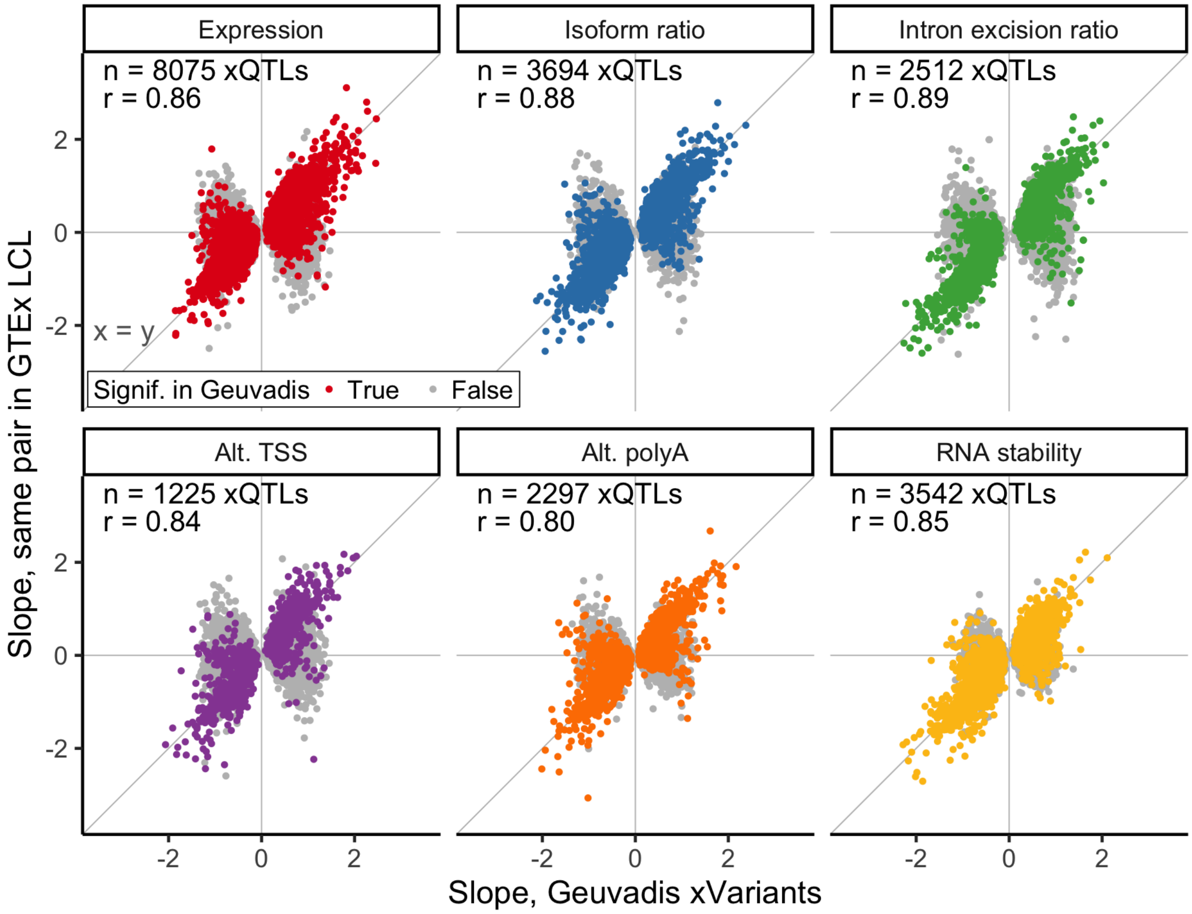

For each modality, we identified the top xQTL association per gene in Geuvadis and extracted the slope of the same variant-phenotype pairs, if tested, from GTEx LCL tissue results. Associations that were significant in Geuvadis are colored according to modality, and nonsignificant associations are gray. Pearson correlation coefficient r was calculated for the significant associations per modality. Gray lines show x = 0, y = 0, and x = y. Source data are provided as a Source Data file.

Supplementary Figure 4: Location of xQTL variants from combined-modality mapping in Geuvadis, relative to exons, introns, and boundaries.

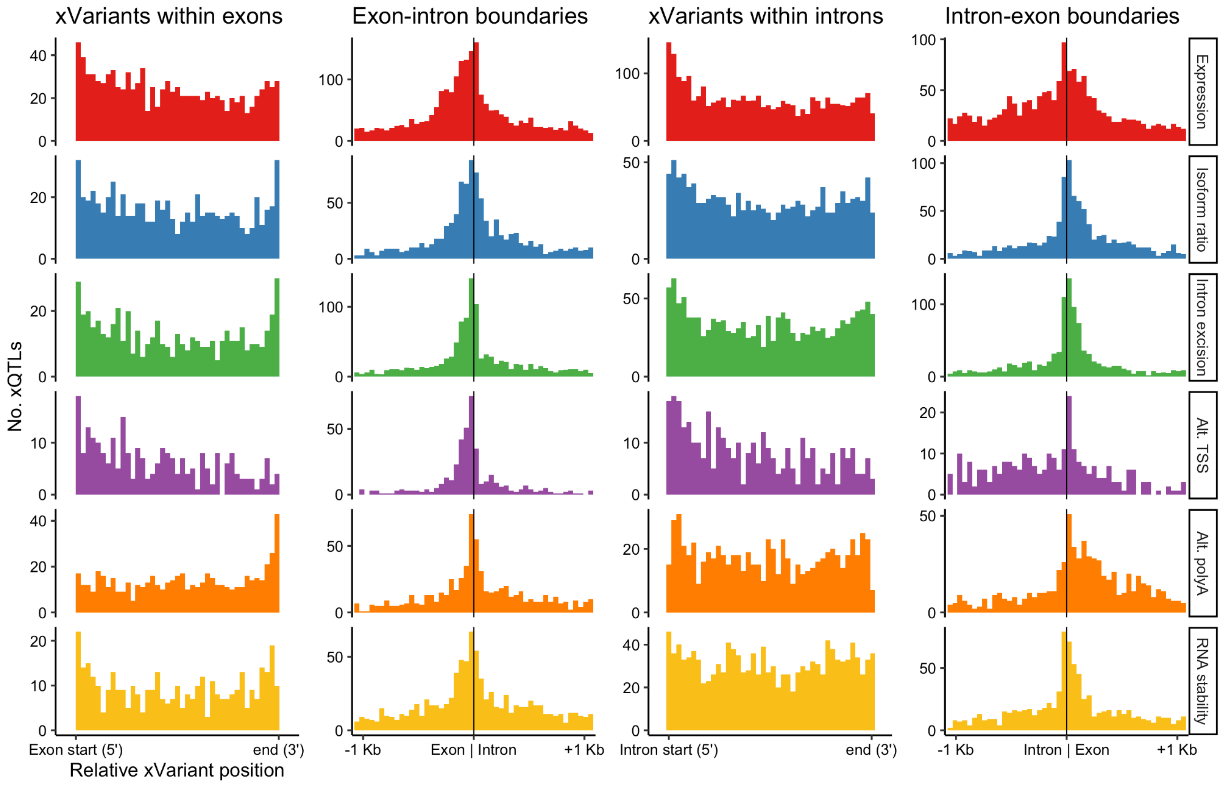

For the first and third columns, the genomic coordinates of xVariants were linearly transformed such that the exon/intron starts and ends were aligned on the x-axis. The second and fourth columns show the distribution of QTL positions within 1 Kb of all exon-intron and intron-exon boundaries, oriented 5’-3’, without normalizing by gene length. Histograms are colored by modality. Source data are provided as a Source Data file.

Supplementary Figure 5: Impact of cross-modality mapping on eQTL effect size concordance.

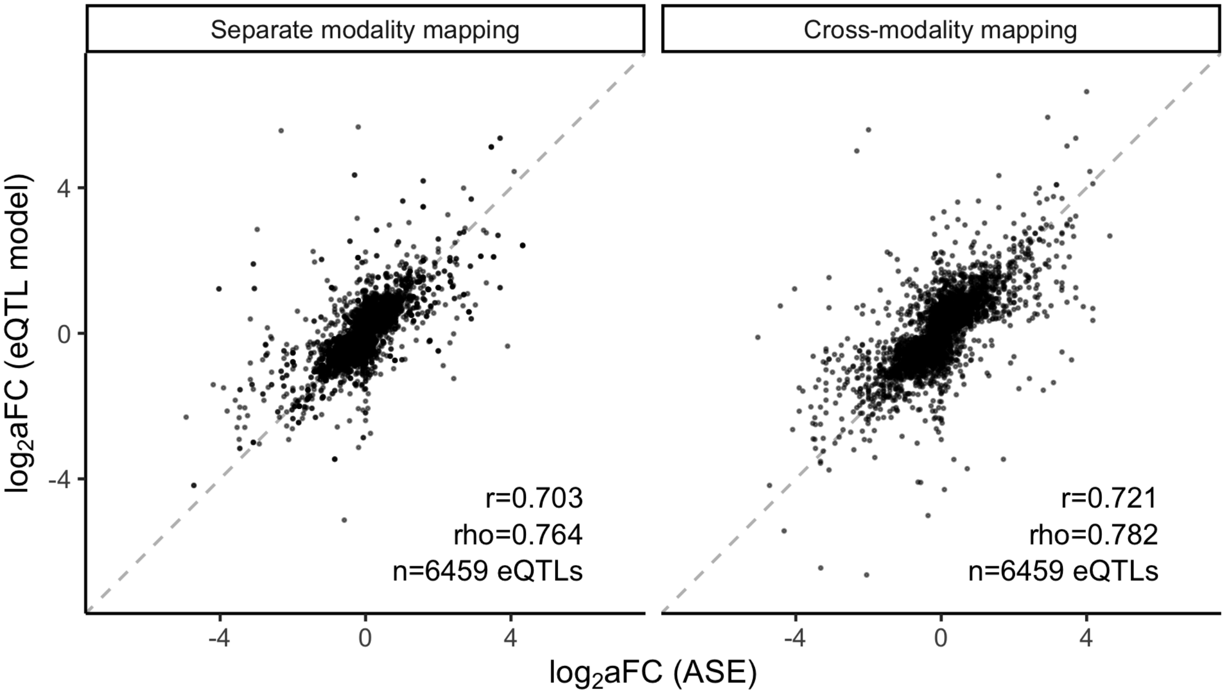

Scatter plots of allelic fold change of eQTLs in subcutaneous adipose GTEx tissue (ADPSBQ), when mapping conditionally independent xQTLs for modalities separately (left) and using cross-modality mapping (right). aFC was measured for each eQTL gene-variant pair using aFC-n (i.e., the eQTL model) and using allele-specific expression (ASE) in heterozygous individuals. For this comparison, eQTL sets were subset and resampled as described in the Methods. Pearson's r and Spearman's rho are shown for both eQTL sets, along with the set sizes. Dashed lines show x = y. Source data are provided as a Source Data file.

Supplementary Figure 6: TWAS colocalization fraction in relation to tissue sample size and number of TWAS hits.

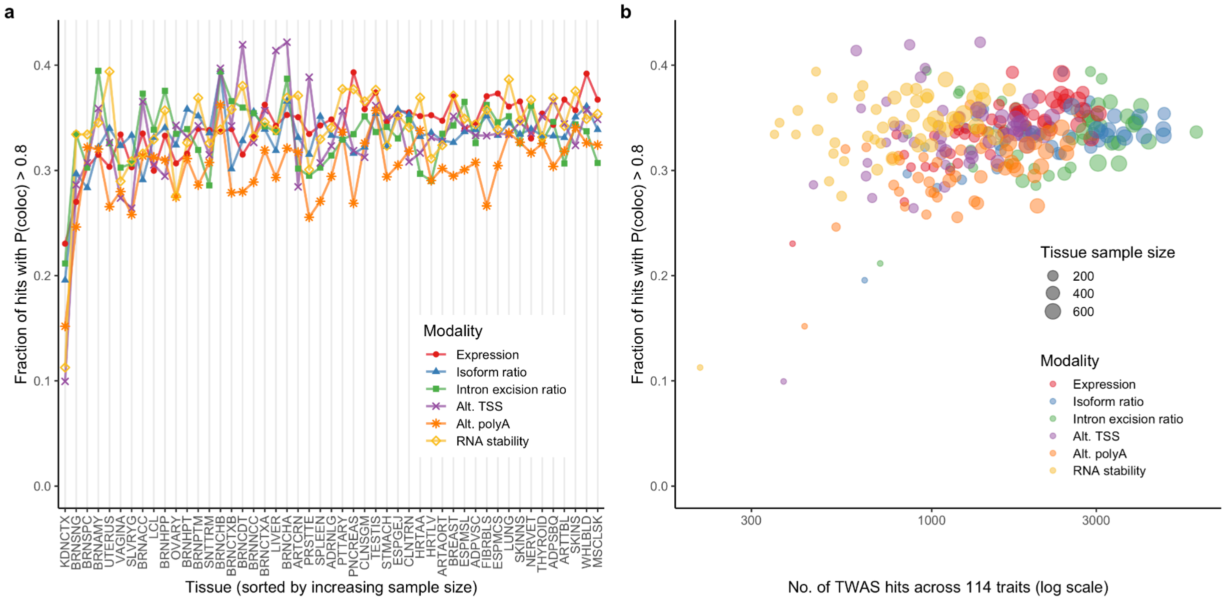

a For each tissue-modality pair, the fraction of TWAS hits across all 114 traits with COLOC PP4 >0.8. Tissues are sorted by increasing sample size. **b** The same colocalization fractions per tissue-modality pair are shown on a scatter plot against the number of TWAS hits, i.e. the denominator of each fraction. Source data are provided as a Source Data file.

Supplementary Figure 7: Modality proportions among top hits per trait-gene pair from FOCUS and FUSION.

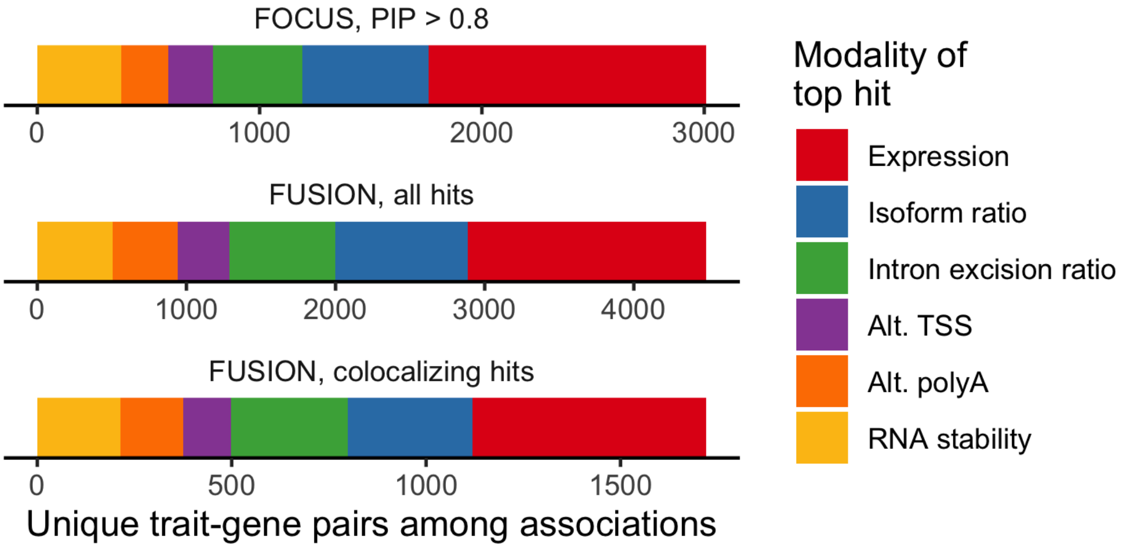

The unique trait-gene pairs represented in the trait-RNA phenotype associations from each method are colored by the modality of the gene's RNA phenotype with the highest posterior inclusion probability (FOCUS) or lowest TWAS p-value (FUSION). PIP, posterior inclusion probability. Source data are provided as a Source Data file.

Supplementary Figure 8: Proportions of RNA modalities in cis-QTLs and TWAS hits.

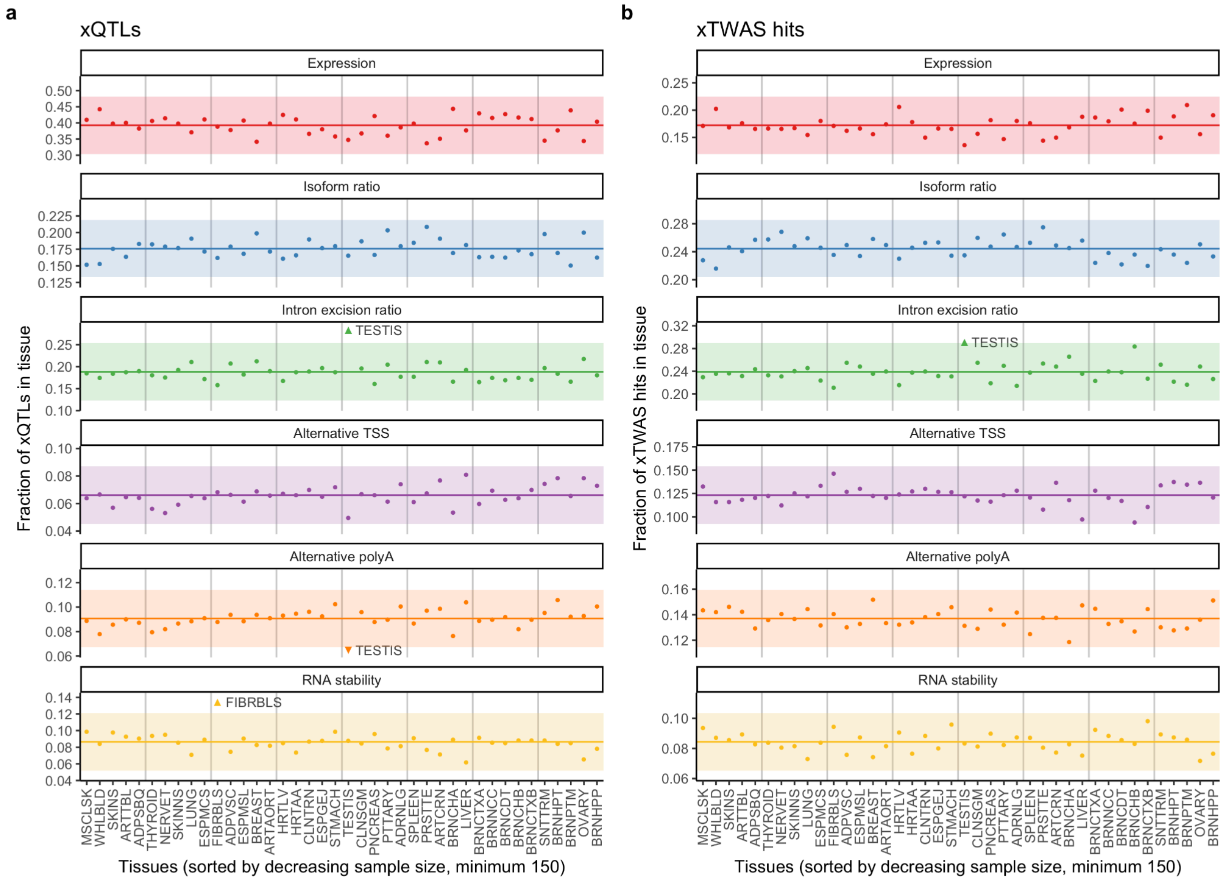

a Fraction of cis-QTLs per modality in each GTEx tissue with sample size >150. Horizontal lines and bands indicate mean and three standard deviations per modality. Upward and downward-pointing triangles indicate values above and below the ±3 standard deviation interval, respectively. **b** The same plot but for the fraction of TWAS hits, across all 114 tested traits. Source data are provided as a Source Data file.

Supplementary Figure 9: Impact on xQTLs of adding sex or age covariate.

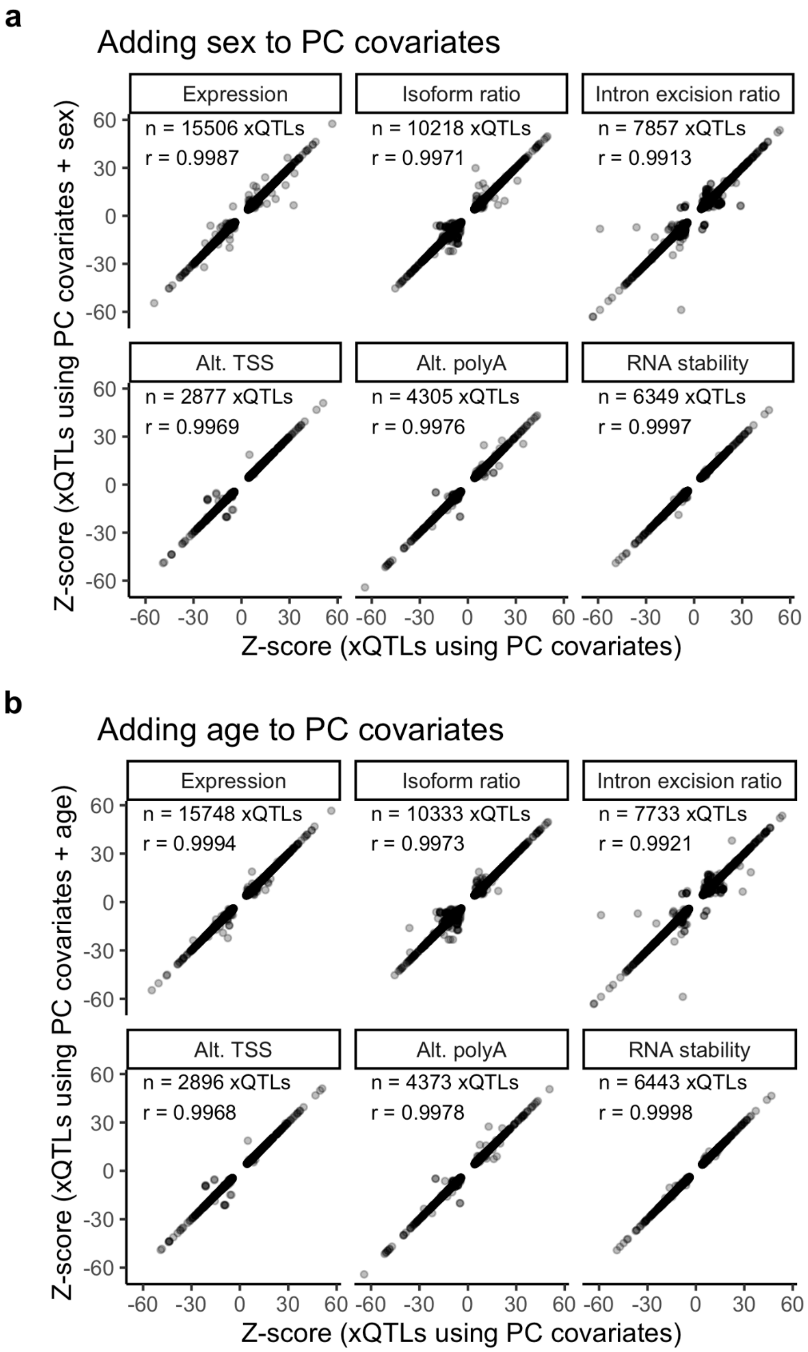

a For each modality separately, xQTLs were mapped as described in the Methods for one GTEx tissue, subcutaneous adipose, and repeated with the addition of sex as a covariate, obtained from GTEx metadata. Scatter plots show the Z-scores of conditionally independent xQTLs per modality, subsetted to the 87.8-88.7% of the no-sex-covariate xQTLs with exact phenotype-top-variant pair matches with sex-covariate xQTLs, and Pearson correlation coefficients are shown. **b** The analysis was repeated with an age covariate in place of sex covariate, also obtained from GTEx metadata. Source data are provided as a Source Data file.

Supplementary Methods

Implementation

The backbone of our Pantry implementation (https://github.com/PejLab/Pantry)[1] is a pipeline built with Snakemake[2]. Snakemake is a workflow management system with an emphasis on bioinformatics pipelines. It provides flexibility both in terms of data and project parameters, which can be specified in configuration files, and in terms of computing environments, such as high-performance computing clusters.

The code for the data processing pipeline consists of existing programs, e.g., STAR[3] and samtools[4], additional scripts to process their input and output data, and the Snakemake code that runs the programs and scripts. All of this pipeline code is contained within a project template directory within Pantry that can be copied, customized, and executed. This allows the pipeline to be customized in terms of parameters or the addition of entirely new phenotypes, while preserving the customized code alongside the data it produces. A second project template directory with similar structure holds the Pheast downstream genetic analysis module.

The default Pantry pipeline was designed for computational and storage efficiency. For example, several modalities involve read quantification with respect to modality-specific annotations. Instead of using a quantification method that requires separate read alignments for each set of annotations, Pantry uses kallisto[5], a pseudoalignment method, for quantification, reducing runtime and greatly reducing intermediate file storage. Similarly, for the BAM alignment files used for other modalities to count intron junctions, exon reads, and intron reads, unused read information is removed from the BAM files, reducing their size by an order of magnitude without affecting the results.

Pantry includes a module called Pheast (PHEnotype Application STreamlined) for running various downstream analyses with all Pantry-generated phenotypes. As with the phenotype-generating code, Pheast is packaged as its own project directory template with a configuration file, Snakemake code, and scripts. It can be copied and edited to run analyses with generated Pantry phenotypes and corresponding genotypes. This two-stage approach allows the complexities of phenotype generation, including reference files, properties of the input sequence data, and the variety of phenotyping software, to be of concern only in the first stage. Then, in the second stage, multiple phenotype sets, formatted uniformly as BED files and accompanying metadata, can be used for each of multiple downstream applications. By default, this stage includes generation of covariates from genotypes and the phenotypes in each set, followed by cis-QTL mapping and fitting xTWAS (FUSION[6]) models.

The Pantry dependency versions used for the analyses in this study are as follows: bedtools v2.30.0[7], gcta v1.93.2[8], htslib v1.14[9], kallisto v0.48.0[5], plink v1.90b6.21[10], plink2 v2.00a3.3[10], regtools v0.6.1[11], samtools v1.15[4], snakemake v7.15.1[2], star v2.7.10a[3], subread v2.0.1[12], tensorqtl v1.0.7[13], FUSION[6], txrevise v2.0[14].

Cross-modality mapping with tensorQTL

Pantry's cross-modality xQTL mapping is implemented using tensorQTL[13] as follows. See https://github.com/broadinstitute/tensorqtl for code and documentation.

1. **Input files**
    a. The `combine_modalities.sh` script in the phenotyping module concatenates the normalized phenotype tables from all modalities into one BED file with unique phenotype IDs.
    b. The script also generates a phenotype groups file mapping each phenotype in the BED file to its gene.
    c. This combined phenotype table is used as input to generate PCs in the same way as for individual modalities, producing 20 phenotype PCs along with the same 5 genotype PCs used for separate modality mapping.
2. **xQTL mapping**
   tensorQTL is run on this input data, along with genotypes, in the same way as mapping one modality that has multiple phenotypes per gene.
    a. That is, tensorQTL is run first in `cis` mode, supplying the phenotype groups file so that all phenotypes assigned to the same gene are processed in a single group.
    b. Then, tensorQTL is run in `cis_independent` mode with the same inputs plus the `cis` mode output. This runs stepwise regression on each group, resulting in one ranked list of conditionally independent cis-QTLs per gene, and any of the gene's phenotypes from any modality could appear zero, one, or multiple times in the list. Both tensorQTL modes employ permutation-based significance testing.

Supplementary References

1. Munro, D. PejLab/Pantry: v1.0.0. Zenodo https://doi.org/10.5281/zenodo.13922024 (2024).
2. Mölder, F. *et al.* Sustainable data analysis with Snakemake. Preprint at https://doi.org/10.12688/f1000research.29032.2 (2021).
3. Dobin, A. *et al.* STAR: ultrafast universal RNA-seq aligner. *Bioinformatics* **29**, 15–21 (2013).
4. Danecek, P. *et al.* Twelve years of SAMtools and BCFtools. *GigaScience* **10**, giab008 (2021).
5. Bray, N. L., Pimentel, H., Melsted, P. & Pachter, L. Near-optimal probabilistic RNA-seq quantification. *Nat. Biotechnol.* **34**, 525–527 (2016).
6. Gusev, A. *et al.* Integrative approaches for large-scale transcriptome-wide association studies. *Nat. Genet.* **48**, 245–252 (2016).

7. Quinlan, A. R. & Hall, I. M. BEDTools: a flexible suite of utilities for comparing genomic features. *Bioinformatics* **26**, 841–842 (2010).

8. Yang, J., Lee, S. H., Goddard, M. E. & Visscher, P. M. GCTA: a tool for genome-wide complex trait analysis. *Am. J. Hum. Genet.* **88**, 76–82 (2011).

9. Bonfield, J. K. *et al.* HTSlib: C library for reading/writing high-throughput sequencing data. *GigaScience* **10**, giab007 (2021).

10. Chang, C. C. *et al.* Second-generation PLINK: rising to the challenge of larger and richer datasets. *GigaScience* **4**, s13742-015-0047–8 (2015).

11. Cotto, K. C. *et al.* Integrated analysis of genomic and transcriptomic data for the discovery of splice-associated variants in cancer. *Nat. Commun.* **14**, 1589 (2023).

12. Liao, Y., Smyth, G. K. & Shi, W. featureCounts: an efficient general purpose program for assigning sequence reads to genomic features. *Bioinformatics* **30**, 923–930 (2014).

13. Taylor-Weiner, A. *et al.* Scaling computational genomics to millions of individuals with GPUs. *Genome Biol.* **20**, 228 (2019).

14. Alasoo, K. *et al.* Genetic effects on promoter usage are highly context-specific and contribute to complex traits. *eLife* **8**, e41673 (2019).
