# Supplementary material for: Multimodal analysis of RNA sequencing data powers discovery of complex trait genetics
Source: Nat Commun. 2024 Nov 29;15:10387. doi: 10.1038/s41467-024-54840-8 (PMC11607376; doi:10.1038/s41467-024-54840-8)
Supplement: Supplementary file 3 — Description of additional supplementary files [file 41467_2024_54840_MOESM3_ESM.pdf]

Description of Additional Supplementary Files

File Name: Supplementary Data 1
Description: Counts of xGenes and xQTLs per GTEx tissue, modalities mapped separately

File Name: Supplementary Data 2
Description: Counts of xGenes and xQTLs per GTEx tissue, cross-modality mapping

File Name: Supplementary Data 3
Description: Counts of xTWAS hits and unique genes per trait-tissue pair for GTEx tissues
