# Supplementary material for: Multimodal analysis of RNA sequencing data powers discovery of complex trait genetics
Source: Nat Commun. 2024 Nov 29;15:10387. doi: 10.1038/s41467-024-54840-8 (PMC11607376; doi:10.1038/s41467-024-54840-8)
Supplement: Supplementary file 7 — Reporting Summary [file 41467_2024_54840_MOESM7_ESM.pdf]

nature portfolio

Corresponding author(s): Daniel Munro

Last updated by author(s): Oct 18, 2024

Reporting Summary

Nature Portfolio wishes to improve the reproducibility of the work that we publish. This form provides structure for consistency and transparency in reporting. For further information on Nature Portfolio policies, see our Editorial Policies and the Editorial Policy Checklist.

Statistics

For all statistical analyses, confirm that the following items are present in the figure legend, table legend, main text, or Methods section.

n/a	Confirmed	
☐	☒	The exact sample size (*n*) for each experimental group/condition, given as a discrete number and unit of measurement
☒	☐	A statement on whether measurements were taken from distinct samples or whether the same sample was measured repeatedly
☐	☒	The statistical test(s) used AND whether they are one- or two-sided<br>*Only common tests should be described solely by name; describe more complex techniques in the Methods section.*
☐	☒	A description of all covariates tested
☐	☒	A description of any assumptions or corrections, such as tests of normality and adjustment for multiple comparisons
☐	☒	A full description of the statistical parameters including central tendency (e.g. means) or other basic estimates (e.g. regression coefficient) AND variation (e.g. standard deviation) or associated estimates of uncertainty (e.g. confidence intervals)
☐	☒	For null hypothesis testing, the test statistic (e.g. *F*, *t*, *r*) with confidence intervals, effect sizes, degrees of freedom and *P* value noted<br>*Give P values as exact values whenever suitable.*
☒	☐	For Bayesian analysis, information on the choice of priors and Markov chain Monte Carlo settings
☒	☐	For hierarchical and complex designs, identification of the appropriate level for tests and full reporting of outcomes
☐	☒	Estimates of effect sizes (e.g. Cohen's *d*, Pearson's *r*), indicating how they were calculated

Our web collection on statistics for biologists contains articles on many of the points above.

Software and code

Policy information about availability of computer code

Data collection	No software was used for data collection.
Data analysis	The Pantry code is available at https://github.com/PejLab/Pantry [https://doi.org/10.5281/zenodo.13922024]. Dependencies used for this study: bedtools v2.30.0, gcta v1.93.2, htslib v1.14, kallisto v0.48.0, plink v1.90b6.21, plink2 v2.00a3.3, regtools v0.6.1, samtools v1.15, snakemake v7.15.1, star v2.7.10a, subread v2.0.1, tensorqtl v1.0.7, FUSION, txrevise v2.0

For manuscripts utilizing custom algorithms or software that are central to the research but not yet described in published literature, software must be made available to editors and reviewers. We strongly encourage code deposition in a community repository (e.g. GitHub). See the Nature Portfolio guidelines for submitting code & software for further information.

Data

Policy information about availability of data

All manuscripts must include a data availability statement. This statement should provide the following information, where applicable:

- Accession codes, unique identifiers, or web links for publicly available datasets
- A description of any restrictions on data availability
- For clinical datasets or third party data, please ensure that the statement adheres to our policy

The data processed with Pantry for Geuvadis and all GTEx tissues are available in a public Zenodo repository [https://doi.org/10.5281/zenodo.13922139]. These include, for all six modalities in each tissue, RNA phenotype matrices, covariates, xQTLs, xTWAS transcriptomic model weights, and xTWAS associations for 114

GWAS traits. This repository is about 42 GB when compressed. Raw Geuvadis data were downloaded from ArrayExpress, accession E-GEUV-1 [https://www.ebi.ac.uk/biostudies/arrayexpress/studies/E-GEUV-1]. Raw protected GTEx data were downloaded from the database of Genotypes and Phenotypes (dbGaP), accession no. phs000424.v8 [https://www.ncbi.nlm.nih.gov/projects/gap/cgi-bin/study.cgi?study_id=phs000424.v8.p2]. GWAS summary statistics used for xTWAS are available at Zenodo record 3629742 [https://doi.org/10.5281/zenodo.3629742]. Figure source data are provided with this paper.

Research involving human participants, their data, or biological material

Policy information about studies with human participants or human data. See also policy information about sex, gender (identity/presentation), and sexual orientation and race, ethnicity and racism.

Reporting on sex and gender	This study reanalyzed existing anonymized data that were not filtered with regard to sex or gender. This study focused on autosomal genetic variation, and sex information was used only in an analysis to measure the impact of using sex as a covariate for genetic analyses.
Reporting on race, ethnicity, or other socially relevant groupings	This study reanalyzed existing anonymized data, and any race, ethnicity, or other socially relevant grouping data previously collected with the human data reanalyzed in this study were not used in these analyses.
Population characteristics	This study reanalyzed existing anonymized data. Principal components from autosomal genotypes were used as covariates to control for genetic confounding. Age information from GTEx was used only in an analysis to measure the impact of using age as a covariate for genetic analyses. Ages for all GTEx individuals ranged from 20 to 70, with first quartile 47, median 55, and third quartile 63.
Recruitment	This study reanalyzed existing anonymized data.
Ethics oversight	This study reanalyzed existing anonymized data.

Note that full information on the approval of the study protocol must also be provided in the manuscript.

Field-specific reporting

Please select the one below that is the best fit for your research. If you are not sure, read the appropriate sections before making your selection.

☒ Life sciences ☐ Behavioural & social sciences ☐ Ecological, evolutionary & environmental sciences

For a reference copy of the document with all sections, see nature.com/documents/nr-reporting-summary-flat.pdf

Life sciences study design

All studies must disclose on these points even when the disclosure is negative.

Sample size	This study reanalyzed existing anonymized datasets and did not collect new samples. The full set of GTEx tissues (the 49 adult tissues used previously by GTEx for genetic analyses) was chosen due to its wide representation of human tissue types, with sufficient sample sizes to demonstrate Pantry's genetic analyses, as evidenced by the results of previous studies using the same GTEx data. We additionally used the Geuvadis dataset as an independent data source to assess replicability of results.
Data exclusions	For the reanalysis of GTEx Project v8 data in this study, the same sample size requirement from the original study was used to determine which tissues were genetically analyzed (>=70 genotyped samples), resulting in the same 49 of 54 tissues genetically analyzed in this study.
Replication	The transcriptomic genetic associations for two biologically similar, independent datasets, Geuvadis and GTEx LCL tissues, were compared. The regression slopes were consistent in both direction and magnitude between the two datasets, with Pearson's correlation coefficients ranging from 0.80 to 0.89 per RNA modality and mean Deming regression slope of 1.005.
Randomization	For each human dataset reanalyzed in this study, individuals were not separated into groups, and no experimental intervention was conducted.
Blinding	Individuals were not allocated into groups in this study.

Reporting for specific materials, systems and methods

We require information from authors about some types of materials, experimental systems and methods used in many studies. Here, indicate whether each material, system or method listed is relevant to your study. If you are not sure if a list item applies to your research, read the appropriate section before selecting a response.

Materials & experimental systems

n/a	Involved in the study
☒	☐ Antibodies
☒	☐ Eukaryotic cell lines
☒	☐ Palaeontology and archaeology
☒	☐ Animals and other organisms
☒	☐ Clinical data
☒	☐ Dual use research of concern
☒	☐ Plants

Methods

n/a	Involved in the study
☒	☐ ChIP-seq
☒	☐ Flow cytometry
☒	☐ MRI-based neuroimaging

Plants

Seed stocks

Report on the source of all seed stocks or other plant material used. If applicable, state the seed stock centre and catalogue number. If plant specimens were collected from the field, describe the collection location, date and sampling procedures.

Novel plant genotypes

Describe the methods by which all novel plant genotypes were produced. This includes those generated by transgenic approaches, gene editing, chemical/radiation-based mutagenesis and hybridization. For transgenic lines, describe the transformation method, the number of independent lines analyzed and the generation upon which experiments were performed. For gene-edited lines, describe the editor used, the endogenous sequence targeted for editing, the targeting guide RNA sequence (if applicable) and how the editor was applied.

Authentication

Describe any authentication procedures for each seed stock used or novel genotype generated. Describe any experiments used to assess the effect of a mutation and, where applicable, how potential secondary effects (e.g. second site T-DNA insertions, mosiacism, off-target gene editing) were examined.
